# Supplementary material for: Noise-augmented directional clustering of genetic association data identifies distinct mechanisms underlying obesity
Source: PLoS Genet. 2022 Jan 27;18(1):e1009975. doi: 10.1371/journal.pgen.1009975 (PMC8794082; doi:10.1371/journal.pgen.1009975)
Supplement: S1 Text — (PDF) [file pgen.1009975.s001.pdf]

Supporting Information for: Noise-augmented directional clustering of genetic association data identifies distinct mechanisms underlying obesity

Andrew J. Grant*[1], Dipender Gill[2,3,4,5], Paul D. W. Kirk[1,6], and Stephen Burgess[1,7]

[1]MRC Biostatistics Unit, University of Cambridge, Cambridge, UK

[2]Department of Epidemiology and Biostatistics, School of Public Health, St Mary's Hospital, Imperial College London, London, UK

[3]Clinical Pharmacology and Therapeutics Section, Institute of Medical and Biomedical Education and Institute for Infection and Immunity, St George's, University of London, London, UK

[4]Clinical Pharmacology Group, Pharmacy and Medicines Directorate, St George's University Hospitals NHS Foundation Trust, London, UK

[5]Novo Nordisk Research Centre Oxford, Old Road Campus, Oxford, United Kingdom

[6]Cambridge Institute of Therapeutic Immunology & Infectious Disease (CITIID), University of Cambridge, Cambridge, UK

[7]Cardiovascular Epidemiology Unit, Department of Public Health and Primary Care, University of Cambridge, Cambridge, UK

*Corresponding author. Email address: andrew.grant@mrc-bsu.cam.ac.uk

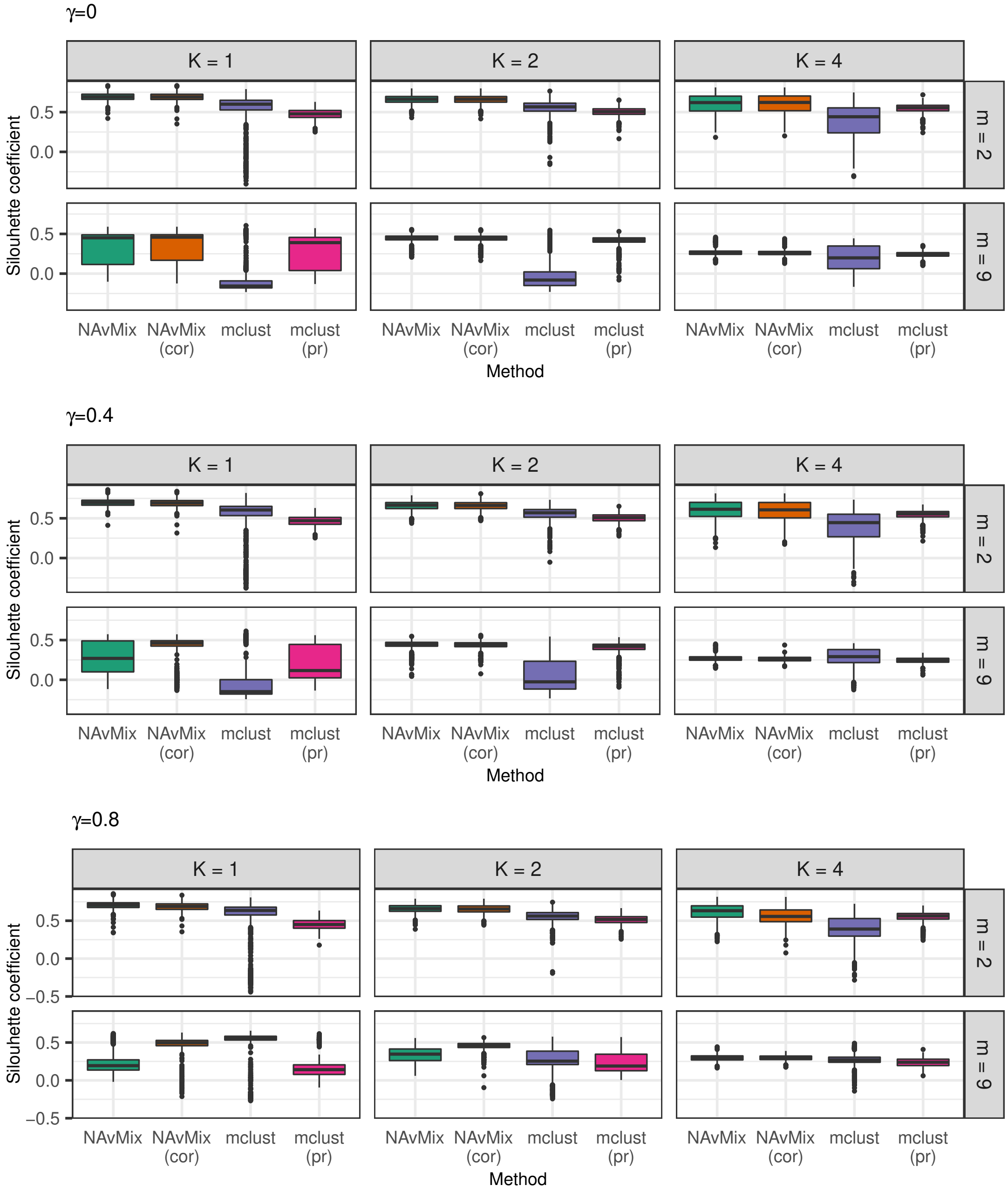

Fig A. Boxplots of the silhouette coefficient for each scenario using NAvMix, NAvMix incorporating trait correlation estimates (cor), mclust, and mclust with proportional effects (pr).

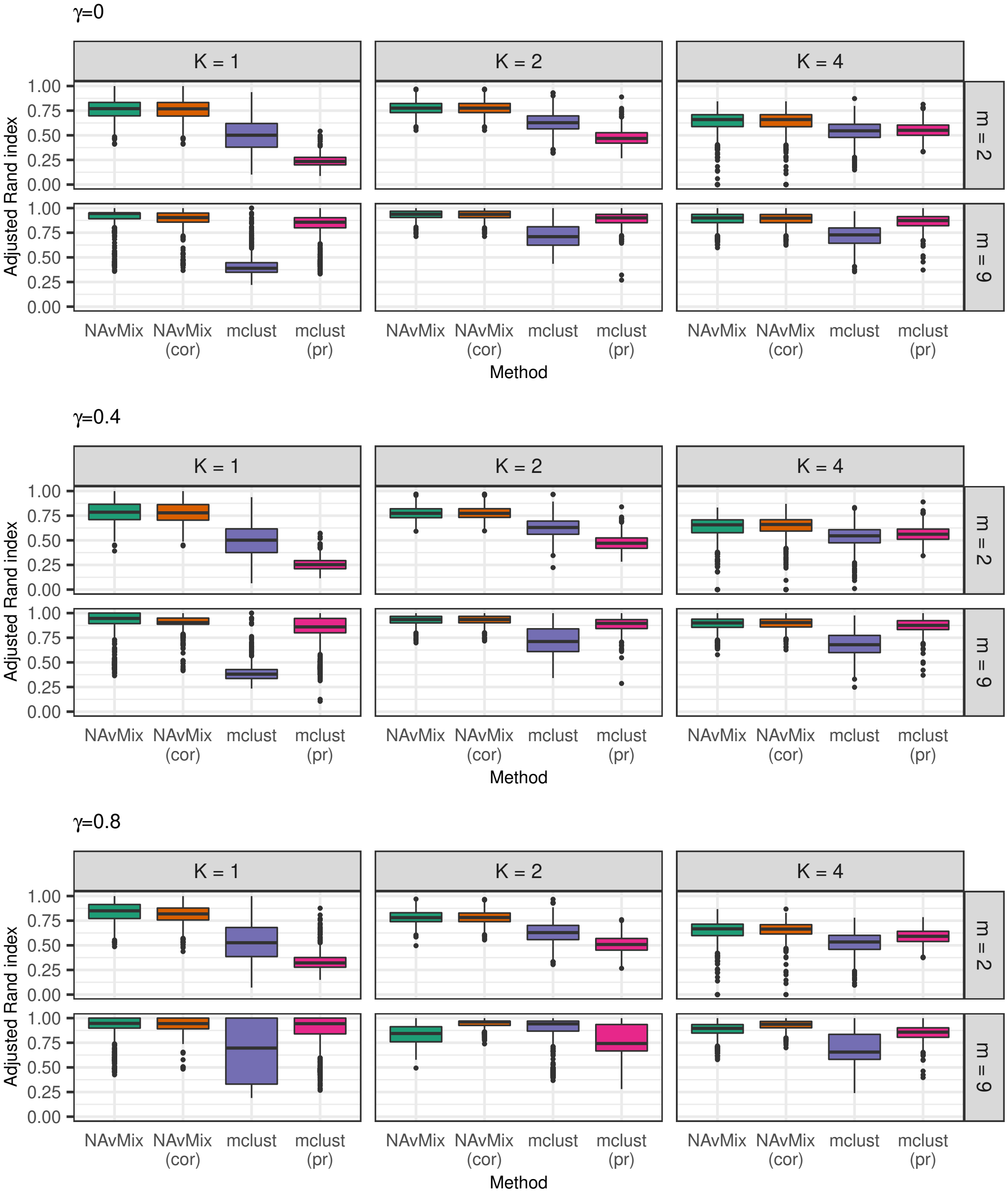

Fig B. Boxplots of the adjusted Rand index for each scenario using NAvMix, NAvMix incorporating trait correlation estimates (cor), mclust, and mclust with proportional effects (pr) when only including genetic variants which were genome-wide significant with respect to at least one trait.

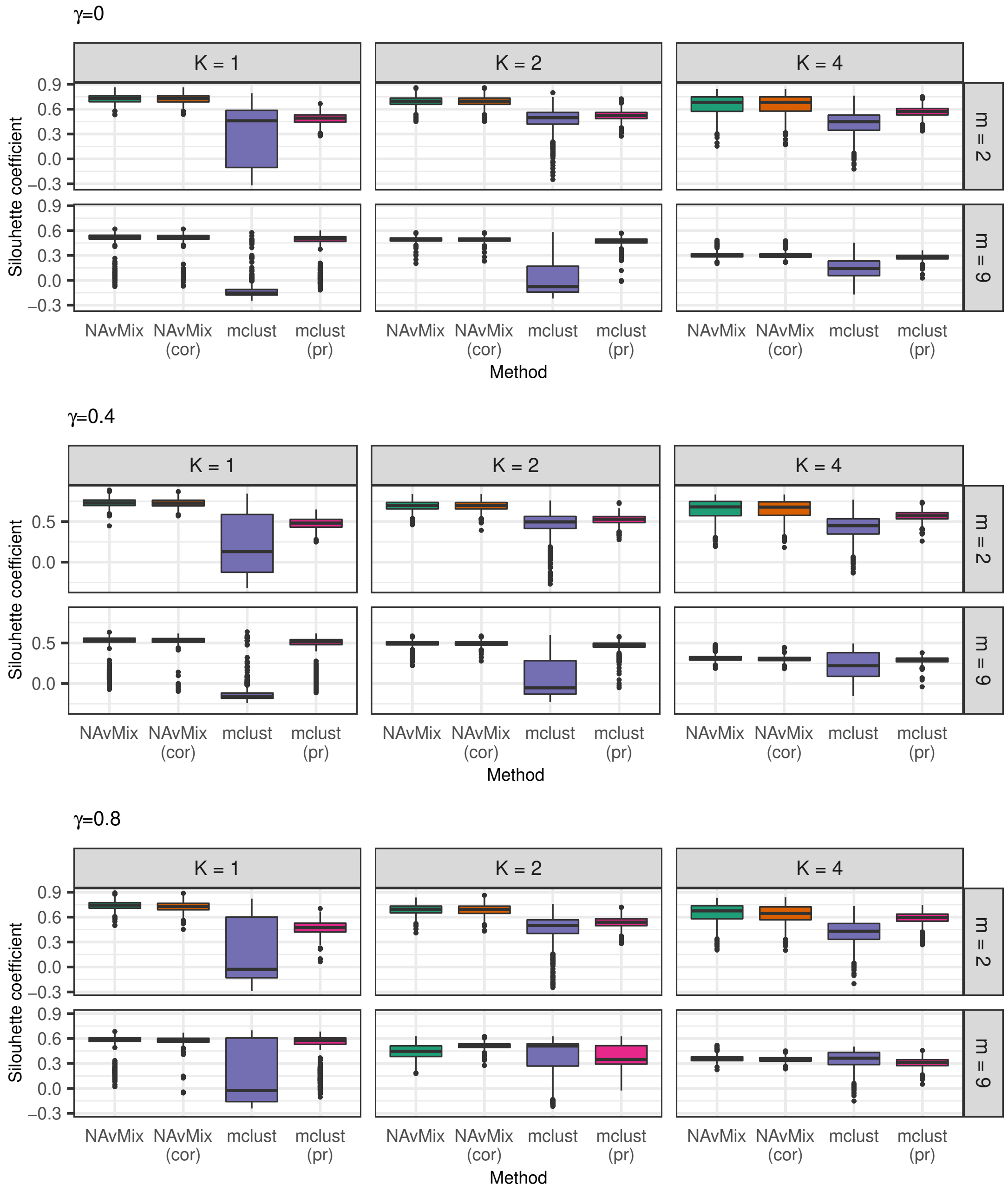

Fig C. Boxplots of the silhouette coefficient for each scenario using NAvMix, NAvMix incorporating trait correlation estimates (cor), mclust, and mclust with proportional effects (pr) when only including genetic variants which were genome-wide significant with respect to at least one trait.

Table A. Mean number of clusters estimated for each simulated scenario using NAvMix, NAvMix incorporating trait correlation estimates (cor), mclust, and mclust using proportional associations (pr) when only including genetic variants which were genome-wide significant with respect to at least one trait.

γ	Number of traits (m)	Number of clusters (K)	Number of clusters			
			NAvMix	NAvMix (cor)	mclust	mclust (pr)
0	2	1	1.01	1.01	1.55	6.70
		2	2.00	2.00	2.13	7.79
		4	3.57	3.57	3.80	7.71
	9	1	1.05	1.03	3.54	1.13
		2	2.01	2.01	4.42	2.03
		4	4.25	4.19	5.16	4.08
0.4	2	1	1.00	1.00	1.65	6.50
		2	2.00	2.00	2.17	7.78
		4	3.53	3.54	3.79	7.74
	9	1	1.07	1.01	3.76	1.20
		2	2.02	2.00	4.26	2.07
		4	4.26	4.06	4.43	4.07
0.8	2	1	1.01	1.02	1.80	6.54
		2	2.00	2.02	2.21	7.36
		4	3.64	3.44	3.83	7.60
	9	1	1.11	1.01	2.78	1.40
		2	2.82	2.00	2.31	3.73
		4	4.31	4.02	3.01	5.08

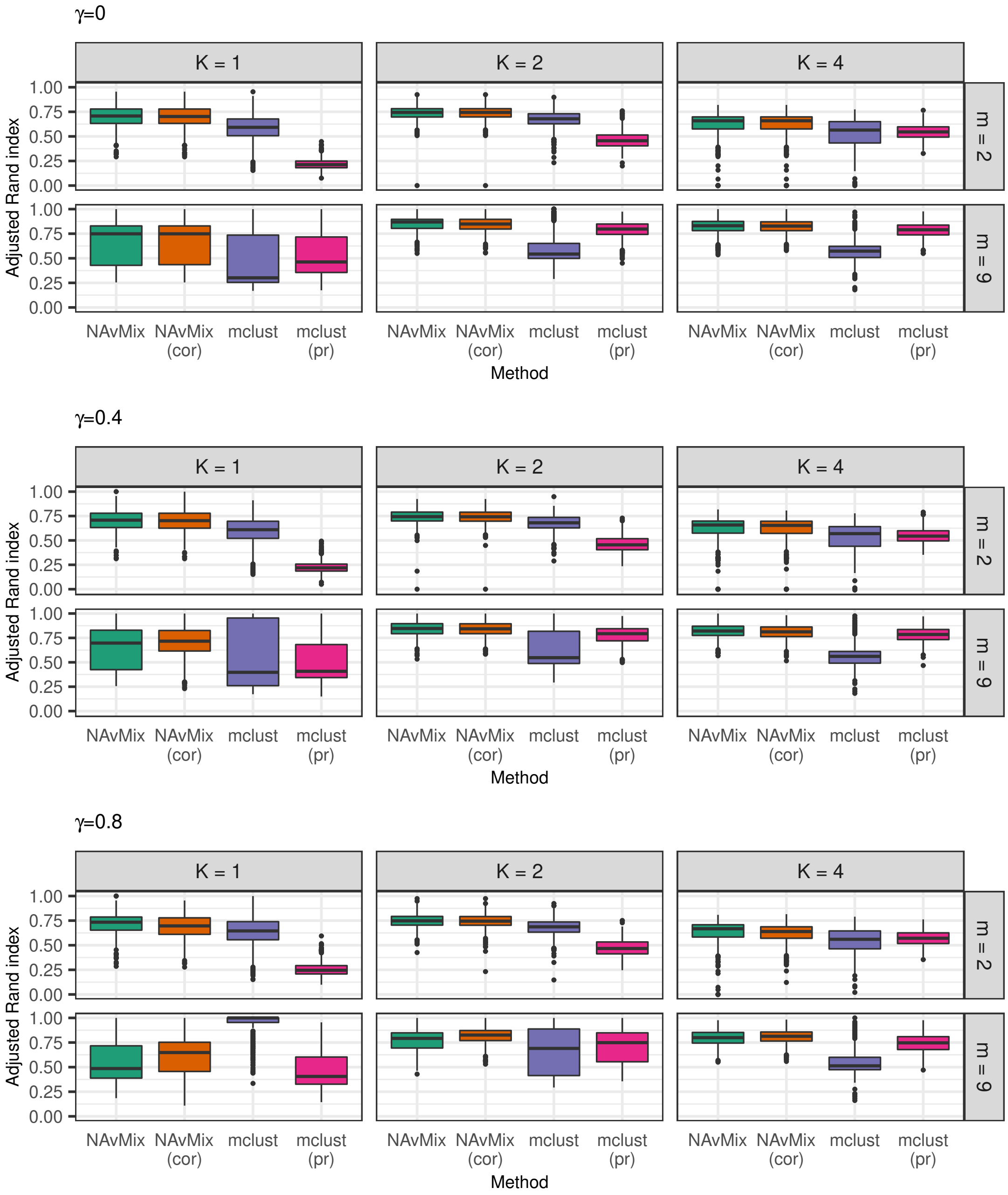

Fig D. Boxplots of the adjusted Rand index for each scenario using NAvMix, NAvMix incorporating trait correlation estimates (cor), mclust, and mclust with proportional effects (pr) when genetic variant-trait associations were estimated in samples of different sizes.

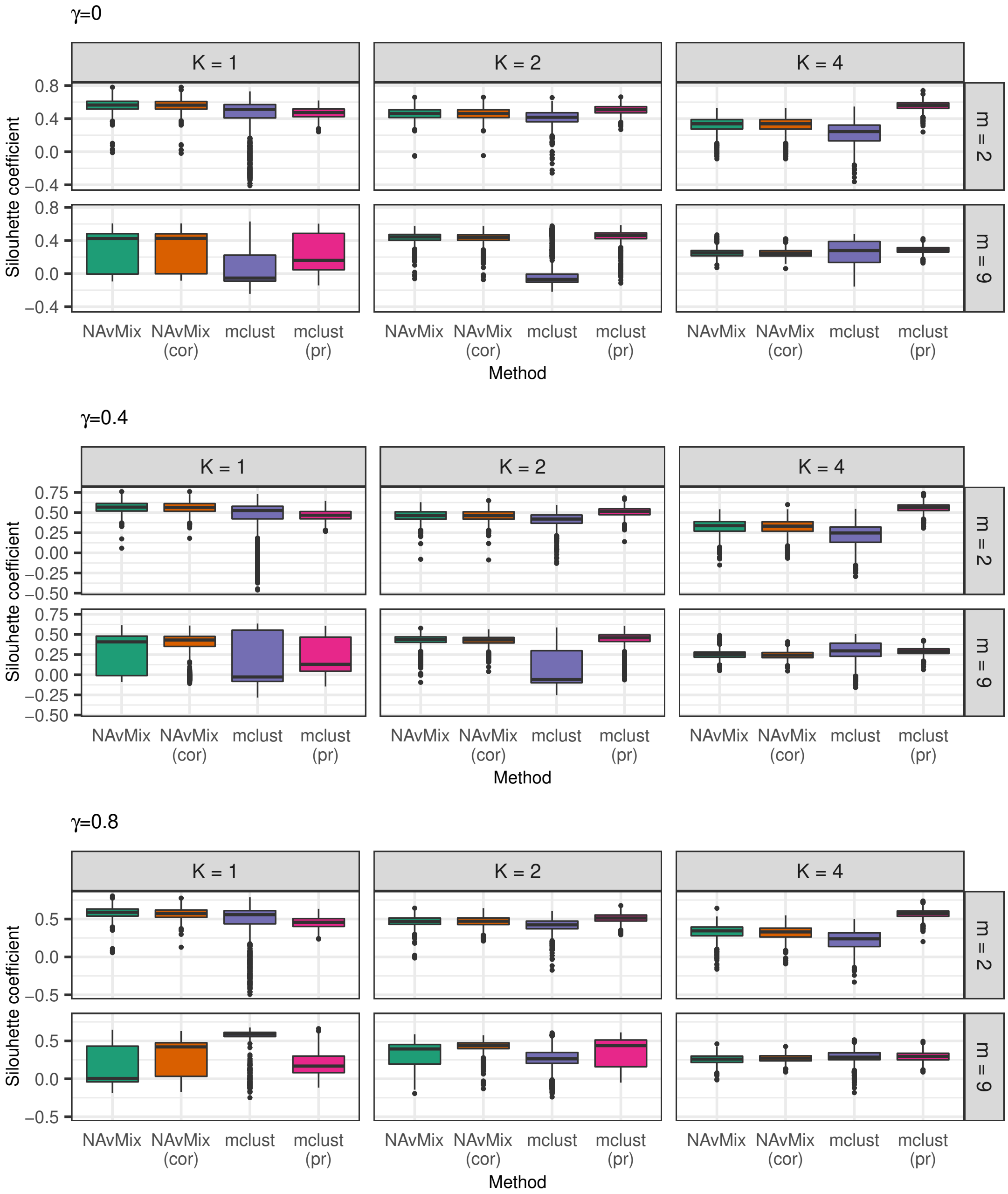

Fig E. Boxplots of the silhouette coefficient for each scenario using NAvMix, NAvMix incorporating trait correlation estimates (cor), mclust, and mclust with proportional effects (pr) when genetic variant-trait associations were estimated in samples of different sizes.

Table B. Mean number of clusters estimated and mean number of observations allocated to the noise cluster for each simulated scenario using NAvMix, NAvMix incorporating trait correlation estimates (cor), mclust, and mclust using proportional associations (pr) when genetic variant-trait associations were estimated in samples of different sizes. The true number of variants in the noise cluster is 20.

			Number of clusters				Number of noise variants			
γ	Number of traits (m)	Number of clusters (K)	NAvMix	NAvMix (cor)	mclust	mclust (pr)	NAvMix	NAvMix (cor)	mclust	mclust (pr)
0	2	1	1.01	1.01	1.23	7.00	19.60	19.70	18.66	9.16
		2	2.02	2.02	2.06	7.94	16.85	16.75	17.36	6.78
		4	3.67	3.68	3.53	8.05	11.20	11.05	13.88	7.38
	9	1	1.47	1.39	3.18	1.69	22.76	23.37	19.61	24.55
		2	2.07	2.06	5.07	2.16	24.98	25.22	19.45	26.94
		4	4.23	4.16	3.42	4.09	23.77	24.59	18.91	27.29
0.4	2	1	1.01	1.00	1.23	6.93	19.84	20.08	18.74	9.43
		2	2.02	2.01	2.05	7.95	16.81	16.97	17.40	6.65
		4	3.67	3.63	3.51	8.08	10.98	12.66	13.89	7.11
	9	1	1.50	1.21	2.70	1.84	22.95	25.09	19.36	24.29
		2	2.08	2.05	4.56	2.25	25.22	25.94	18.75	26.97
		4	4.23	4.06	2.90	4.09	24.09	25.99	18.51	27.49
0.8	2	1	1.01	1.03	1.27	6.60	20.36	20.54	18.62	10.41
		2	2.03	2.04	2.06	7.90	16.79	16.36	17.38	7.04
		4	3.74	3.47	3.48	7.97	10.25	16.71	13.86	7.61
	9	1	1.90	1.31	1.12	2.08	22.28	25.79	19.49	24.13
		2	2.77	2.07	2.21	3.18	24.37	26.35	18.55	25.30
		4	4.32	4.02	2.48	4.78	25.07	26.39	18.88	27.30

Table C. Intercept estimate, standard error of estimate (SE), 95% confidence interval (CI) and p-value from the MR-Egger intercept test when examining the association of genetically predicted BMI with CHD, for all genetic variants and for each cluster.

Instruments	Estimate	SE	CI	p-value
All genetic variants	-0.002	0.002	(-0.006, 0.001)	0.135
Cluster 1	-0.002	0.002	(-0.006, 0.002)	0.335
Cluster 2	0.002	0.002	(-0.002, 0.007)	0.358
Cluster 3	-0.002	0.005	(-0.011, 0.007)	0.657
Cluster 4	0.006	0.007	(-0.009, 0.020)	0.442
Cluster 5	-0.022	0.015	(-0.050, 0.007)	0.133

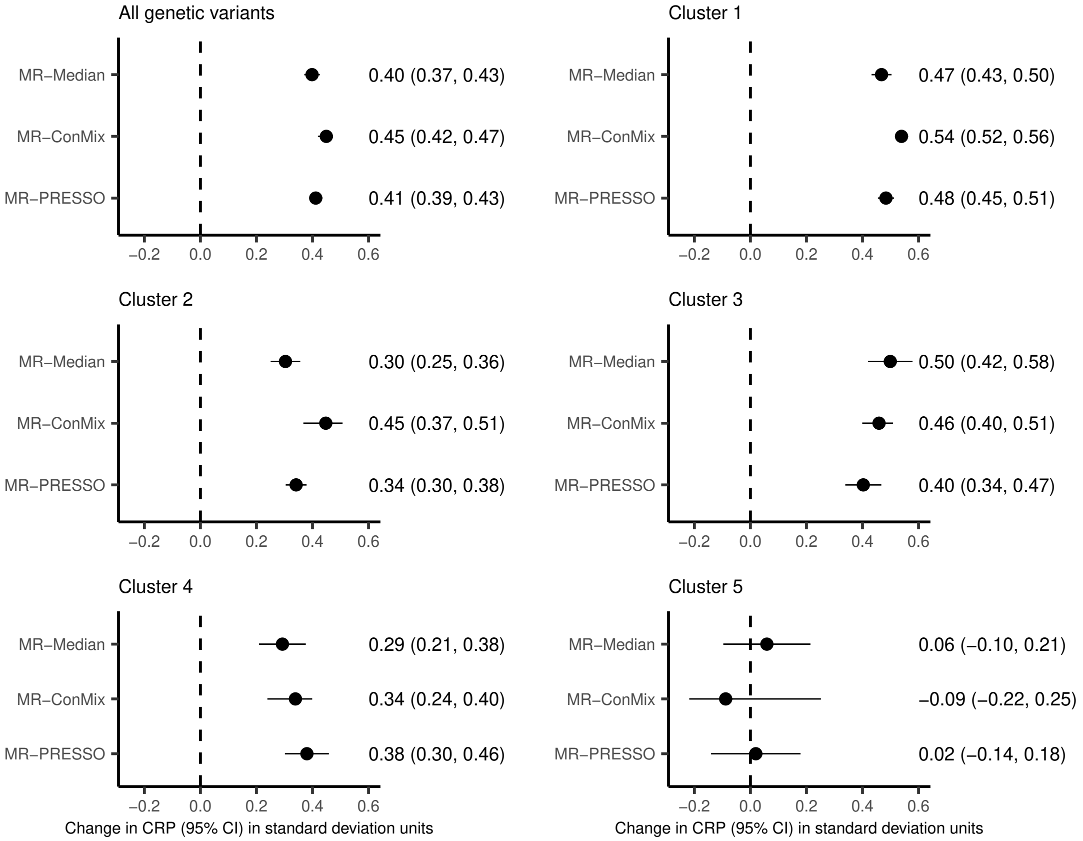

Fig F. Estimates and 95% confidence intervals of the association of genetically predicted BMI with CRP from MR-Median, the Contamination Mixture method (MR-ConMix) and MR-PRESSO, for all genetic variants and for each cluster. Estimates represent the change in CRP in standard deviation units per 1 standard deviation increase in genetically predicted BMI.

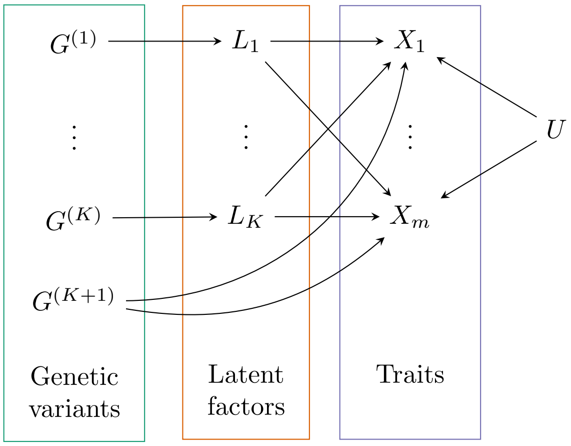

Fig G. Directed acyclic graph showing the relationship between the genetic variants, K latent factors, m traits and confounding variable U in the simulation study.

Table D. List of cytokines and growth factors

Abbreviation	Full name
βNGF	Beta nerve growth factor
CTACK	Cutaneous T-cell attracting
EOTAXIN	Eotaxin
FGF Basic	Basic fibroblast growth factor
GCSF	Granulocyte colony-stimulating factor
GROα	Growth regulated oncogene-α
HGF	Hepatocyte growth factor
IFNγ	Interferon-gamma
IL1B	Interleukin-1-beta
IL1ra	Interleukin-1 receptor antagonist
IL2	Interleukin-2
IL2rα	Interleukin-2, alpha subunit
IL4	Interleukin-4
IL5	Interleukin-5
IL6	Interleukin-6
IL7	Interleukin-7
IL8	Interleukin-8
IL9	Interleukin-9
IL10	Interleukin-10
IL12p70	Interleukin-12p70
IL13	Interleukin-13
IL16	Interleukin-16
IL17	Interleukin-17
IL18	Interleukin-18
IP10	Interferon gamma-induced protein 10
MCSF	Macrophage colony-stimulating factor
MCP1	Monocyte chemotactic protein-1
MCP3	Monocyte specific chemokine 3
MIF	Macrophage migration inhibitory factor (glycosylation-inhibiting factor)
MIG	Monokine induced by interferon-gamma
MIP1α	Macrophage inflammatory protein-1α
MIP1β	Macrophage inflammatory protein-1β
PDGFbb	Platelet derived growth factor BB
RANTES	Regulated on Activation, Normal T Cell Expressed and Secreted
SCF	Stem cell factor
SCGFβ	Stem cell growth factor beta
SDF1α	Stromal cell-derived factor-1 alpha
TNFα	Tumor necrosis factor-alpha
TNFβ	Tumor necrosis factor-beta
TRAIL	TNF-related apoptosis inducing ligand
VEGF	Vascular endothelial growth factor

Table E. Sample sizes for the GWAS in which genetic associations with each trait were estimated as well as the number of the 539 BMI associated genetic variants which are associated with each trait at the genome-wide significance level (Sig. variants).

Trait	Sample size	Sig. variants
Body mass index	806,834	539
Body fat percentage	361,194	179
Systolic blood pressure	361,194	12
Triglycerides	361,194	27
HDL-cholesterol	361,194	29
Educational attainment	293,723	8
Physical activity	91,105	2
Lifetime smoking score	462,690	12
Waist-to-hip ratio	697,734	117
Type 2 diabetes	298,957	26

Table F. List of BMI associated genetic variants excluded from Mendelian randomization analyses due to them not being available in the outcome dataset

Outcome(s)	Cluster	rsid
CHD	1	rs79113395
	1	rs7774
	3	rs10761785
CRP	3	rs10761785
Cytokines & growth factors	1	rs79113395
	1	rs77165542
	1	rs16851483
	1	rs150215901
	1	rs6950388
	1	rs10110189
	1	rs118081010
	2	rs17024393
	2	rs3770890
	2	rs2962334
	2	rs16906838
	2	rs76638898
	3	rs13107325
	3	rs116374395
	3	rs10761785
	3	rs1126930
	3	rs4812405
	4	rs74887628
	4	rs79780963
