# Supplementary material for: Noise-augmented directional clustering of genetic association data identifies distinct mechanisms underlying obesity
Source: PLoS Genet. 2022 Jan 27;18(1):e1009975. doi: 10.1371/journal.pgen.1009975 (PMC8794082; doi:10.1371/journal.pgen.1009975)
Supplement: Attachment — Submitted filename: Response to Reviewers.docx [file pgen.1009975.s005.docx]

**Response to Reviewer Comments for: Noise-augmented directional clustering of genetic association data identifies distinct mechanisms underlying obesity**

We would like to express thanks to the Reviewers and Editors for their time and helpful comments on our manuscript. We have responded to each comment below. Reviewer comments are in black and are numbered for reference. Our responses are in blue. We have also provided a marked version of the main manuscript where all changes are coloured in blue.

**Reviewer: 1**

The manuscript by Grant and colleagues proposed a novel method for clustering genetics variants based on their associations with multiple related traits. The advantage of cross-trait analysis, compared with single-trait analysis, is that it can potentially partition variants underlying a trait of interest into multiple clusters/patterns, each associated with a distinct mechanism. The manuscript proposed a novel approach to variant clustering, based on directions of variant effects on multiple traits. This is an interesting and well-motivated idea and is a timely contribution to this field. Applying this method, NAvMix, the authors reported some interesting findings in BMI and related traits.

We thank the Reviewer once again for their helpful and constructive comments.

Major comments

**A1**

(1) One major issue of cross-trait analysis is overlapping samples between different studies. This is potentially a serious problem, as it would induce correlations of variant effects across traits, a pattern potentially mis-identified as true clusters. The proposed strategy to deal with this seems inadequate. Most often, individual level data is not available, and according to the authors, NAvMix, will simply set the off-diagonal terms in the covariance matrix 0, i.e. ignoring the possible correlations. The justification is that this is not a problem in simulations. However, it's possible that in the simulation setting, the correlations induced by shared samples are too weak, compared with the correlation of true effect sizes. As a result, ignoring induced correlations does not change the results much. This may not be the case, however, in the real data.

My suggestion is: better justification using more extensive simulations. In particular, it would be helpful to vary the relative importance of true effect size correlations vs. correlation induced by shared samples. It would be helpful, for example, to vary how much phenotypic variations are explained by genetic variants vs. by the shared underlying factors ($U_i$, in simulations). Related to this point: it would be helpful to better explain some of the parameter settings in simulations. Ex. effect sizes are drawn from certain distributions. Are the parameters of these distributions realistic?

Another suggestion: it is possible to estimate correlation of effect sizes between traits, due to shared samples, using cross-trait LD score regression [PMID: 26414676]. That paper uses full GWAS summary statistics to estimate genetic correlation, while accounting for shared samples. It shows that the "residual" correlation due to shared samples is a simple function of phenotypic correlations, the sample sizes of the two studies, and the number of shared samples. In fact, it can directly estimate this residual correlation. It may be possible to directly use these estimates (at an appropriate scale) in NAvMix.

Many thanks for these, and other, suggestions regarding the simulation study. We have revised the simulation study according to your suggestions made in this comment, as well as comments A3, B3, B4 and C2. The changes are outlined in detail in the Methods section. In brief, we now generate the genetic variants which belong in the substantive clusters as associating with one of K latent factors. These latent factors act on the traits in a way which represents a distinct mechanism. For example, in the case with two traits and two clusters, one latent factor acts on both traits positively, while the other acts positively on trait 1 and negatively on trait 2. Genetic variants belonging to the noise cluster act directly on the traits.

The genetic variant associations with the latent factors, and the direct genetic associations in the case of the noise variants, have been chosen such that the proportion of variance in each trait explained by the genetic variants is approximately 5-10%. They are also generated such that, for a given trait, most of the variants will be only weakly associated with the trait, with a small number which are highly significant. We believe the chosen parametrisation is realistic for a setting where a small to moderate number of traits have been chosen which are believed to be relevant for clustering the genetic variants according to their biological mechanisms.

We systematically vary the proportion of the variance in the traits explained by the shared variable U_i by varying the \gamma parameter. We perform the simulations for \gamma = 0, 0.4 and 0.8. When \gamma = 0, the traits are uncorrelated. This also proxies the setting where the traits may be correlated but measured in separate samples. Thus, increasing \gamma shows the effect of increased trait correlation and/or sample overlap. In the figures showing the simulation results, we include results from NAvMix both with and without trait correlation estimates.

As the results show, the method of incorporating trait correlation estimates into the estimated covariance matrices produces similar results to the approach where these correlation estimates are ignored, in all but the settings with very high trait correlation (\gamma = 0.8) and a higher dimensional set of traits (m = 9). This is a fairly extreme example, in that the traits are all highly correlated with each other and in the same direction. In practice, the chosen trait set is likely to be more curated than this, as we have suggested in the Discussion. Nonetheless, as an extra supplementary analysis, we repeated the clustering on the simulated datasets using only genetic variants which have at least one genome-wide significant association with the traits. This improves the results in the cases with high trait correlation.

We believe that these new results justify our proposed approach of incorporating trait correlation estimates if they are available. We also believe that, unless the traits are thought to be highly correlated, it is reasonable to proceed as though they are not correlated (that is, by setting the off-diagonal matrices of the covariance matrices to zero), as we have done in the applied example. We suggest in the Discussion that if there are traits which are highly correlated, then domain knowledge should be used to select only one of them (since this does not add much extra information and may impact the results if trait correlation estimates are not available). Furthermore, we have included a comment in the Discussion proposing to consider using cross-trait LD score regression for the case where the chosen traits are highly correlated, there is substantial sample overlap, and individual data are not available.

*…the simulation study suggested that, unless the traits are highly correlated with each other, the method is robust to ignoring the genetic variant-trait association correlations. This also suggests that the approach is robust to some participant overlap in the samples. If the traits are highly correlated, there is significant sample overlap, and individual level data are not available, a potential alternative approach accounting for trait correlation is using the intercept term from cross-trait LD score regression [40]. These estimates could then be incorporated into the* $\hat{\Sigma}_{j}$ *matrices.*

**A2**

(2) The noise cluster presumably increases the robustness of the method. However, in the real data, only 1 out of >500 BMI variants is assigned to the noise cluster. One possible explanation is that: the mean of the noise cluster is the global mean direction. This may be too similar to the major clusters (clusters 1 and 2 in the BMI results). As a result, not many variants would be assigned to the noise cluster. It seems to make sense to use a broader distribution (uniform at some scale) for the noise cluster.

The von Mises-Fisher distribution with kappa = 0 is equivalent to the uniform distribution on the (m-1)-dimensional unit sphere. Thus, the choice of mean vector in the noise cluster is arbitrary, and we have chosen to input the global sample mean for convenience. We have added extra comment in the Methods section to clarify this point.

*Note that, since the noise cluster is uniformly distributed, the value of* $\mu_{K+1}$ *is arbitrary, and we choose the global sample mean for convenience.*

Although applying NAvMix in the applied example resulted in only 1 variant in the noise cluster, we do not expect that so few observations will be considered as noise in general. Furthermore, as per S2 Table, a number of other variants were given non-zero probability of belonging to the noise cluster, and so if a thresholding approach to cluster membership was taken, more variants would be pulled into the noise cluster.

**A3**

(3) The simulation procedure largely follows the model of NAvMix. So perhaps unsurprisingly, NAvMix performs well under those simulations. A more realistic, or biologically motivated simulation would give a better idea of the performance of NAvMix. The authors could explicitly simulate a few "latent factors", which act on the observed phenotypes. Then each variant could either act on these latent fators, or act on some traits directly. I think if each trait acts only on one factor a time, then this reduces to the variant clustering problem. The advantage of this simulation regime is that one could easily vary some of the parameters and assess if NAvMix are robust to these changes. For example, is the method robust to direct effects of variants (i.e. variants acting on a trait without affecting any of the factors)?

Once again, thank you for your suggestions regarding the simulation study. We have now revised the simulation study according to this, and other, reviewer comments (see A1, B3, B4 and C2). The clusters in the revised simulations are formed according to latent factors, with variants associating directly on the traits acting as noise variants. This setup is illustrated in the directed acyclic graph in Fig. G in the Supporting Information. Full details on the revised simulation study are given in the Methods section, and a brief overview in the response to A1.

**A4**

(4) The main difference of clusters 1 and cluster 2 is that cluster 1 variants are associated with smoking, but cluster 2 not. I found this result interesting/puzzling. Given the smoking association, I would imagine that cluster 1 may capture some "behavior" component of BMI. Indeed, it was reported that hertiability of obesity is mostly enriched in brain, and it is not hard to imagine that smoking variants act through some kind of additive behavior. However, the pathway analysis of cluster 1 does not suggest anything related to behavior/brain. One concern is that the results may be driven by sample sharing between BMI and smoking GWAS, though it's not clear to me if this would lead to different clusters. In any case, it would be helpful to check this - could be done using the intercept term of cross-trait LDSC, as mentioned above. In fact, it would be good to check the correlations driven by shared samples for all pairs of traits.

Another relevant question is: has BMI GWAS data adjusted smoking, and vice versa? Is it possible that associations of BMI variants with smoking driven by some kind of collider bias? This could happen when one has a causal model like this: SNP -> smoking <- additive behavior -> BMI. Adjusting smoking in GWAS of BMI may lead to false association of SNP and BMI.

Thank you for this insightful observation. There are two possible explanations for the difference in smoking association between Clusters 1 and 2: one, as you have suggested, is that it is driven by addictive behaviour; another is that it is driven by some other mechanism entirely.

We have looked at the associations between Cluster 1 and 2 variants and alcohol intake frequency, another addiction-related trait. For Cluster 1 variants, the mean Z-score is 1.55 (with 8 variants associated at genome-wide significance), and for Cluster 2 variants, the mean Z-score is 0.92 (with 0 significant variants). In comparison, the mean Z-score for smoking is 2.38 for Cluster 1 variants (with 9 significant variants) and -0.46 for Cluster 2 variants (with 0 significant variants). Although there is some difference in association strength with alcohol intake between the clusters, it is much closer than with the smoking associations. Furthermore, as you have pointed out, there are no addictive behaviour related pathways associated with Cluster 1 variants which were identified by the gene set analysis, even when this was expanded to include GO analysis (see A9). This would all suggest that it may be a non-addictive behaviour related mechanism which is driving the difference in smoking association between these two clusters. We are reluctant to speculate in too much detail without a more comprehensive analysis, however we have added the following comment around this in the Discussion.

*Interestingly, variants in Clusters 1 and 2 were similar in their average associations across each of the traits considered with the exception of smoking, where Cluster 2 had close to zero association. One possible explanation for this is that these variants differ according to some addictive behaviour related mechanism. However, no such pathways were identified in the gene set analysis for Cluster 1. This suggests that some other mechanism may be driving this change, although further analysis is required to identify what this may be.*

The BMI GWAS is not adjusted for smoking, or vice-versa, so collider bias should not be a problem. Indeed, all traits used for clustering are non-adjusted for BMI. Furthermore, because BMI is not a trait used in the clustering trait set, correlation between it and the other traits used (for example, from sample overlap) should not affect the clustering. We have included a comment around the proposal for using cross-trait LDSC regression to estimate correlation between the clustering traits in the Discussion (see also A1).

**A5**

(5) The authors suggest that cluster 5 variants represent some kind of "favorable adiposity", that reduces inflammatory cytokines and hence the CHD risk. This is an attractive model. However, I would caution against over-interpretation. The data basically says that cluster 5 variants have effects on inflammatory cytokines. It's unclear that this effect is mediated through favorable/protective adiposity. It's possible that these variants have some pleiotropic effects on both BMI and cytokines, without adoposity playing any role in the regulation of these cytokines. To rigorously make this claim, I'd imagine some mediation type of analysis, where one controls BMI or some measures of favorable adiposity, and show that the cluster 5 variants would be no longer associated with the CHD risk. I doubt such data is available for this type of analysis though.

Many thanks for this comment. Indeed, as you have suggested, a mediation analysis would be difficult to perform. We have therefore moderated our language in the Discussion and Abstract around suggesting that inflammation mediates the effects of BMI on CHD. Instead, we say that the evidence suggests that inflammation may be related to the heterogeneity between the effect of the different components of genetically predicted obesity on CHD.

*By analysing the biological pathways underpinning the different clusters, we found evidence supporting that the heterogeneity of the effects of the different genetically predicted components of obesity on cardiovascular risk may be related to inflammation.*

Minor comments

**A6**

- Variants in cluster 4 have metabolic functions, and are associated with HDL and TG. However, it is not found to have an effect on CHD risk in MR analysis. This is a bit unexpected, given that LDL is a known risk factor of CHD. It would be good to examine the LDL association of cluster 4 variants.

Recent evidence supports ApoB, which is highly correlated to LDL-cholesterol, as the deterministic lipid fraction for CHD (Ference et al., 2019; Richardson et al., 2020; Zuber et al., 2020). Thus, varying HDL and triglycerides, without affecting LDL-cholesterol would not necessarily affect CHD risk. Our results show that Cluster 4 variants are, on average, associated with increased HDL (mean Z-score of 1.14) and decreased triglycerides (mean Z-score of -1.75). Using data from UK Biobank, we considered the association of the variants with LDL-cholesterol (trait LDL direct). Cluster 4 variants are, on average, associated with decreased LDL-cholesterol (mean Z-score of -1.45). These results are consistent with Cluster 4 variants having no affect, or possibly even some protective effect, on CHD risk, as shown in our MR analysis (OR of CHD per increased SD unit of Cluster 4 predicted BMI was 0.94).

Ference, BA, et al. Association of triglyceride lowering LPL variants and LDL-C-lowering LDLR variants with risk of coronary heart disease. JAMA. 2019; 321(4):364-373.

Richardson, TG, et al. Evaluating the relationship between circulating lipoprotein lipids and apolipoproteins with risk of coronary heart disease: A multivariable Mendelian randomisation analysis. PLoS Med. 2020; 17(3): e1003062.

Zuber, V, et al. High-throughput multivariable Mendelian randomization analysis prioritizes apolipoprotein B as key lipid risk factor for coronary artery disease. Int J Epidemiol. 2020; 50(3):893-901.

**A7**

- Typo: line 314, norm of $x_j$ = 1 for all $i$ - I suppose it should be "for all j".

Thank you, this has been corrected.

**A8**

- Typo: denoted $X_{ik}$ for individual i and trait l, in line 406.

Thank you, this has been corrected.

**A9**

- Table S5: it would be helpful to show the fold enrichment (or expected number of overlapping genes). Also, the authors used KEGG and REACTOME. It would be helpful to add GO analysis as well, which is more commonly used for this type of analysis, and may cover more biological processes.

We have added to Table S5 (now S3 Table) the expected number of overlapping genes as well as results from GO analysis.

**Reviewer: 2**

The authors have proposed a new procedure NAvMix (and its R implementation) to cluster variants based on their genetic effects on multiple traits. The procedure considers a separate cluster for noise on top of the existing clustering method on the unit hypersphere based on a mixture of von Mises-Fisher distributions. The authors have shown good performance of NAvMix in simulations. The authors have applied NAvMix on genetic variants associated with BMI to unravel distinct biological pathways. However, I have some concerns with the results. The simulation study is somewhat narrow in scope (e.g. their data generating mechanism appears close to the main feature of their procedure that they are leveraging; a wider range of evaluation metrics could have been considered). Takeaways from different real data analyses are not always clear. I have provided detailed comments below.

We thank the Reviewer for their helpful and constructive comments.

**Major Comments**

**B1**

1. “Assuming all genetic associations are estimated with the same sample size for a given trait, this will not distort the direction vector. Otherwise, the direction vector will be weighted toward traits for which the associations are more precisely estimated.” - Is this a limitation of the method? What would be practical solution to this issue? Are the authors recommending using NAvMix on traits with similar sample size only? When working with summary statistics on different traits, it is often the case that sample sizes differ widely. For e.g. recent meta-analysis [PMID 34059833] of glycemic traits included ~90K samples for 2hr glucose whereas >280K samples for fasting glucose.

This statement is referring to the case where genetic associations for different variants are estimated with different sample sizes for the same trait. Although this is not uncommon in GWAS, the differences would not normally be so great that this would cause a problem. Nonetheless, if there are significant differences, the unstandardised genetic associations may be used as inputs, possibly as a sensitivity analysis alongside the standardised associations. We have re-worded this statement to be clearer on the point we are making.

*Assuming all genetic associations are estimated with the same sample size for a given trait, this will not distort the direction vector. If there are significant differences between sample sizes used to estimate genetic associations for the same trait, and associations with different traits are on similar scales, the unstandardised association estimates may also be used, possibly as a sensitivity analysis.*

In terms of different sample sizes for different traits, although we considered this originally in a supplementary simulation study, we have now considered this scenario in our expanded simulation study with much larger disparity in sample sizes. The simulations now select sample sizes which are up to five times different (between 10,000 and 50,000). The results are in line with those of the primary simulation study (see Figures D and E and Table B in the Supporting Information), suggesting that this level of disparity does not pose a problem with the clustering approach.

**B2**

2. Lines 68-69: Genetic variants for which vector of association estimates are input in the algorithm are assumed to be independent of each other. Do the authors recommend that only the top hit in a locus be included in the set to be clustered? But then, for most traits, only a handful of loci are significant (not anywhere close to the number 100 assumed in the simulations or the 539 variants in the their real data analysis— a huge number that one can get only from very large sample sizes like they have used). Can this method be used for maybe 10-20 independent variants or on traits with modest sample sizes? Do the authors recommend a lower limit to these numbers?

Genetic variants in the same gene region that are highly correlated through linkage disequilibrium would be expected to associate very similarly with respect to a set of traits, and so we do not believe it would be informative to include variants which are not independent. Even if considering only uncorrelated genetic variants, there are still a large number of traits for which GWAS have found 100+ significant genetic variants, and this will only grow as GWAS sample sizes increase.

Certainly, it would be difficult to find meaningful clusters out of a very small number of variants, particularly if the set of traits is moderately large. For cases where only a small number of variants are genome-wide significant with the primary trait of interest, we would suggest lowering the significance threshold for inclusion, rather than including correlated variants. We have included the following comment in the Discussion.

*In the applied example, the genetic variants used for clustering were chosen according to them being associated with a primary trait of interest, in this case BMI. This resulted in a fairly large number of variants to cluster, in part because of the very large sample size of the GWAS in which these associations were estimated. Other traits of interest may not have so many independent variants associated with them at genome-wide significance. A low number of variants may make it more difficult to find true clusters if the cluster sizes are small. Nonetheless, there are many traits for which, say, 100 or more variants have been found to associate, and this will only grow as GWAS sample sizes increase. Furthermore, the simulation results showed that our clustering approach is still generally able to detect relatively small clusters, with clusters as small as 10 variants out of 100 in total in some settings. In the case where there are only a very small number of variants associated with the primary trait of interest, we would recommend lowering the threshold for inclusion below genome-wide significance rather than include correlated variants. Genetic variants which are not independent would be expected to associate similarly with the given traits, and so it would not be informative to include these.*

**B3**

3. The data generating mechanism (DGM) for the simulations seems somewhat favorable for the “directional statistics” concept that the authors are leveraging in their procedure. In my opinion, it will be useful to consider more realistic and a different DGM. For e.g., instead of using angles between genetic effects to generate different clusters of effects, the authors can consider a structural equation model (as their DGM) reflecting different DAGs capturing the different underlying biological mechanisms among traits (for different DAGs the authors may follow Fig 1 of PMID 29226067)

Thank you for your suggestions regarding the simulation study. We have revised the simulation study in accordance with your comments and those of the other reviewers (see also A1, A3, B4 and C2). We have described the new simulation setup in detail in the Methods section and illustrated the chosen DGM with a DAG in the Supporting Information (see Fig. G). See also the response to A1 for a brief overview of the revised simulation study.

**B4**

4. Clustering evaluation metrics: The authors considered Rand Index. Isn’t adjusted Rand index a better metric to use since it is adjusted for chance? What about additionally using other popular metrics (like normalized mutual information, Silhouette index, etc) that capture other aspects of clustering that Rand index cannot? For evaluation, I think considering metrics that do not require a priori knowledge of clusters is also important.

Thank you for these suggestions. In our expanded simulation study we evaluate the results using four metrics: Adjusted Rand index (which measures how well the clustering method allocated observations to the true cluster, similar to the normalized mutual information); silhouette coefficient (which indicates how well the observations fit within their allocated cluster, and does not require a priori knowledge of the true clusters); the mean number of clusters found; and the mean size of the noise cluster. Overall, we believe that this range of metrics now gives a comprehensive evaluation of the proposed method.

**B5**

5. Fig 5: I see that different clusters of genetic instruments may or may not give different causal relation result. I don’t see a clear distinction between clusters in terms of their MR results. Can the authors clarify the takeaway from the MR analysis done here?

The Mendelian randomization analysis is performed to estimate the effect of the different components of BMI predicted by the various clusters on CHD risk. Overall, as shown in the top row of Figure 5, increased genetically predicted BMI increases the risk of CHD, in line with the established literature. The components of BMI predicted by Clusters 1, 2, and 3 show a similar effect size and direction to the overall result. However, the result using Cluster 5 variants is very different to the first three clusters. Mendelian randomization suggests that an increase in the component of BMI predicted by Cluster 5 variants decreases CHD risk, with non-overlapping 95% confidence intervals compared with the estimates from the other four clusters. We believe this is quite an interesting result, and motivated our further analysis looking at biological pathways and, subsequently, the effects of inflammation on CHD. We have commented on these findings in more detail in the Discussion section.

**B6**

6. The required input/variables/format for their R package is not clear. What parameter choices do users need to make when using NAvMix package? It will be useful for readers if the authors can provide an outline of how readers can implement their procedure from start to finish.

We have created expanded documentation to go with the R package, including extra functionality for standardising the inputs to the NAvMix algorithm. This documentation is in the form of a vignette available on the GitHub page along with the R code.

**Minor Comments**

**B7**

7. Line 149: Are the authors including SNPs with r^2<0.001? If not, I am not sure why Lines 68-69 say that variants should not be in LD.

Although, in theory, we assume that the variants are perfectly in linkage equilibrium, in practice it may be difficult to find variants that satisfy this. Thus, we prune according to a very low r^2^ threshold of 0.001, so that any correlation between variants is extremely low.

**B8**

8. Lines 332-333: specify the contents of parameter vector \Theta.

The \Theta vector has been specified when first used (line 366).

**B9**

9. There might be some notational inconsistency here and there. For e.g., what do X_j, X_k stand for in Line 398 and are they related to vector x_j in the previous paragraphs? Is vector x_j =\hat\Sigma_j^{-1/2} \hat\beta_j? It is worth clarifying these relations for readers.

Thank you for pointing these out, we have corrected these inconsistencies.

**B10**

10. What happens to \pi_k estimate if there are only a few members in a cluster (other than the noise cluster)? Will the estimate be unstable? Can unstable estimates for such a cluster lead to lack of convergence?

In our revised simulation study, we have included a cluster with only 10 out of 100 variants to show results for a case where the true cluster size is small. In the m=2 case, NAvMix tends to underestimate the number of clusters slightly. However, this is not the case in the m=9 case. If the size of one cluster was very small, it would be difficult for any clustering method to detect. However, it should not cause instability in the algorithm, rather these variants would tend to be incorporated into the noise cluster. We have included some comment around very small true clusters being a limitation in the Discussion (see also B2).

**B11**

11. Figs 3-7: It will be helpful to have the number of SNPs in each cluster mentioned in the figures.

Thank you for this suggestion. We have indicated the number of variants in each cluster in Fig. 3.

**B12**

12. Figs 6 & 7: Only MR-IVW results are presented. MR-IVW is least likely to be robust to violation of assumptions (it is unlikely that all 539 SNPs are valid instruments). Is only MR-IVW presented because the results for these MR analyses using different methods/assumptions give the same qualitative results?

Sensitivity analyses for the MR results in Fig. 6 are given in Fig. F in the Supporting Information. All results are in line with the MR-IVW estimates. We have now also included the results from these same sensitivity analyses applied to the cytokine analysis (see Table S4). The results of these sensitivity analyses are in line with the MR-IVW results.

**Reviewer 3**

This paper aims to answer the question ‘how do genetic variants influence complex traits?’ and presents a new approach for clustering genetic variants according to the direction of association with complex traits and incorporates a noise model to improve overall clustering results. They perform simulation studies and apply their method to studying variants associated with high BMI and look at the effects on coronary heart disease.

The paper introduces a novel model but needs more extensive benchmarking and robustness tests to convince me of its utility.

We thank the Reviewer for their helpful and constructive comments.

Major Concerns:

**C1**

I’d like to see some justification/evidence that the magnitude of associations to traits is not important, but the direction of association is the key concern. Is there biological rationale for claiming that magnitudes of association do not encode useful information about how variants influence traits?

The idea that the direction of associations with traits is informative of biological mechanisms has been discussed in several papers. For example, Winkler et al. [2] considered the associations of genetic variants with BMI and WHR, and classified them into four groups: +ive associations with both traits; -ive associations with both traits; +ive associations with BMI and -ive associations with WHR; -ive associations with BMI and +ive associations with WHR. Our clustering approach uses the same underlying idea to cluster variants, but does so in a data-driven way which can be extended to higher dimensional settings. Similarly, Yaghootkar et al. [9] and Udler et al. [3] also discuss that similar directionality of genetic associations relates to shared mechanisms. Although the latter two references apply clustering methods, their methods are based on clustering variants according to Euclidean distances. Our new approach therefore links the concept of searching for common directional association patterns to a clustering method which is directly based on this metric.

We have added reference to this previous literature in the Introduction to show that the concept of directionality has been previously considered. We have also explicitly commented that this approach to defining mechanisms implicitly assumes linearity of the relationships between the genetic associations with the traits.

*Relating similar direction of genetic associations to shared biological mechanisms has been discussed by, for example, Yaghootkar et al. [9], Winkler et al. [2] and Udler et al. [3]. We note that implicit in this way of defining mechanism is the assumption that the relationships between the genetic associations with one trait and the genetic associations with each of the other traits are linear.*

**C2**

Benchmarking should be done over a wider range of comparison models and over parameter values (Why were the number of traits chosen to just be 2 or 9?) I’d also like to see answers to questions about limitations of the model - (How many data points are required to detect clusters reliably? What is the smallest fraction that the method can feasibly detect? How does performance change as a function of noise?)

The main feature of our clustering approach in comparison to existing ones is that it clusters based on direction of association, rather than on the Euclidean distance between observations. Many methods which have been previously used for these applications cluster according to the latter, for example hierarchical clustering, nonnegative matrix factorization and k-means. Although these approaches all differ from each other in some ways, we would expect their results in our simulation study to be broadly similar. We have chosen Gaussian mixture models as the main comparison method because this method is able, through the mclust package, to incorporate some of the features of NAvMix, namely the inclusion of a noise cluster and an automatic way of choosing the number of clusters. It is therefore the most competitive model to ours and serves as a proxy for Euclidean distance-based clustering approaches generally. We use mclust in two ways: using standardised association estimates as inputs; and using the proportional association estimates as inputs. The latter demonstrates the effect of applying a method which clusters based on Euclidean distance to directional data.

The dimension of the trait set may be a relevant factor in the performance of the clustering approach, and so we considered low dimensional (m = 2) and moderate dimensional (m = 9, in line with the applied example) scenarios. Although we do not wish to place limits on this dimensionality, we have commented in the Discussion section that scenarios with a moderate to large number of traits, many of which are not relevant to the clustering, are beyond the scope of this paper and the subject of future research.

In the revised simulation study, we explore the effect of changes in trait correlation and sample sizes, and we consider scenarios where a cluster includes only 10 observations out of 100 in total, including 20 noise observations. NAvMix generally does well in detecting this smaller cluster in the m = 9 scenarios. In the m = 2 scenarios, it detected between 3.3 – 3.7 clusters, on average, suggesting that this smaller cluster was sometimes not picked up.

The specific number of observations required to detect a cluster would depend on many factors, for example: the number of traits; the number of genetic variants; the size of the noise cluster relative to the size of the substantive clusters. Although it is unfeasible to systematically vary every one of these in our simulation study, we believe our revised simulation study provides a comprehensive evaluation of the method, and potential limitations are noted in the Discussion section.

Moderate Concerns:

**C3**

I’d like to see a justification for choosing a uniform noise model. Does incorporating this noise model improve performance over simple pre-processing/data cleaning approaches?

I’d like a brief explanation for why they believe that the other approaches they benchmark achieve lower Rand index on their simulations. Why were these models selected for benchmarking?

A uniform distribution for a noise cluster has commonly been used in Gaussian mixture model clustering since proposed by Banfield and Raftery [43]. Using the uniform distribution over the unit sphere is therefore a natural approach for our directional clustering paradigm, and is computationally easy and efficient to implement due to its relationship with the vMF distribution. This approach was also shown to perform well in the simulation study. Nonetheless, the model-based approach we use would allow for alternative noise distributions, and this could be explored as part of future work. We have included the following comment around this in the Methods section.

*The use of a uniform distribution for a noise cluster has been commonly used in Gaussian mixture models [43], and our model gives a directional analogue of this approach. Alternative approaches to incorporating a noise component to Gaussian mixture models have also been proposed [44, 45, 46]. Although beyond the scope of the present work, different noise distributions for NAvMix could be explored by changing the density of component K + 1.*

The lower Rand index achieved by the Gaussian mixture model approaches is in large part because these methods are designed to cluster on Euclidean distance instead of direction of association. This is illustrated in Fig. 1. Furthermore, applying these approaches to the association vectors normalised to have unit length (that is, the proportional associations) would not be expected to perform well, since the normalised vectors will not be Gaussian, even if the underlying observations are. As we note in the Results section, the only alternative clustering packages available which use a directional clustering approach (e.g., skmeans [14] and movMF [15]) cannot estimate the number of clusters and/or incorporate a noise cluster, and so do not provide a fair comparison.

**C4**

Is the assumption of no linkage disequilibrium a fair assumption?

Genetic variants in the same gene region that are highly correlated through linkage disequilibrium would be expected to associate very similarly with respect to a set of traits, and so we do not believe it would be informative to include variants which are not independent. For cases where only a small number of variants are genome-wide significant with the primary trait of interest, we would suggest lowering the significance threshold for inclusion, rather than including correlated variants. We have added comment around these issues in the Discussion (see also B2).

Minor Concerns:

**C5**

Figure 4 is a bit confusing - can this be better illustrated? The figure makes it appear that several clusters are actually quite similar - are the differences between them significant?

We have replaced this figure with the same information in a parallel plot which we believe now presents this clearer.

**C6**

Figure 5,6 - What do the dotted lines represent?

We have updated the captions in these figures to explain what the dotted lines represent (namely, the regression line from the MR-IVW method in Figure 5 (left column), indicating an odds ratio of 1 in Figure 5 (right column), and indicating no association between genetically predicted levels of CRP and BMI in Figure 6).
