# Supplementary material for: Noise-augmented directional clustering of genetic association data identifies distinct mechanisms underlying obesity
Source: PLoS Genet. 2022 Jan 27;18(1):e1009975. doi: 10.1371/journal.pgen.1009975 (PMC8794082; doi:10.1371/journal.pgen.1009975)
Supplement: Attachment — Submitted filename: Response to Reviewers.docx [file pgen.1009975.s006.docx]

**Response to Reviewer Comments for: Noise-augmented directional clustering of genetic association data identifies distinct mechanisms underlying obesity**

We would like to express thanks once again to the Reviewers and Editors for their time and helpful comments on our manuscript. We have responded to each comment below. Reviewer comments are in black and are numbered for reference. Our responses are in blue. We have also provided a marked version of the main manuscript where all changes are coloured in blue.

**Reviewer 2**

We thank the Reviewer for their further suggestions to improve the manuscript.

1. Table 1: In the caption or somewhere appropriate, it will be useful to provide the actual number of noise variants in the simulated data to better appreciate the estimated “Number of noise variants” from each method.

We have added this information to the caption of Table 1 (also to the caption of Table B in S1 Supporting Information).

2. Lines 153-154: The authors say “This suggests that, unless there is substantial trait correlation or sample overlap, the procedure is robust to missing these estimates.” Since the difference in adjusted Rand index between NAvMix and NAvMix (cor) increases quite a bit with increasing number of traits, decreasing number of clusters, and increasing strength of correlation (similar observation for silhouette index in Fig A), the authors need to include these other conditions in the above statement.

We have added an extra comment after this line to point out the increasing importance of incorporating trait correlation estimates, in the case where traits are correlated, as the number of traits increase and the number of true clusters decrease.

“*This suggests that, unless there is substantial trait correlation or sample overlap, the procedure is robust to missing these estimates. Incorporating trait correlation becomes more important as the number of traits increases and the number of true clusters decreases.”*

3. Regarding this correlation: Authors discuss limitations of NAvMix, which includes “It uses as input the full covariance matrix of the genetic variant-trait associations.” However, I am not sure why this is a limitation. Under reasonable assumptions, the authors approximate \Sigma_j as in lines 450-451. While individual-level data readily gives Corr(X_k, X_l), one can also estimate this correlation easily and quickly using summary statistics only. The field of cross-phenotype association tests using summary statistics uses such an estimate all the time (see for e.g. PMID 29226385). I believe the authors can use such an estimate instead of making the sub-optimal assumption that off-diagonal elements of \Sigma_j are 0.

We agree that the full covariance matrices can be estimated, either using individual or summary level data, under fairly reasonable assumptions. To be conservative, we have left the requirement of full covariance matrices listed as a limitation, since it is not immediately obtained from typical GWAS output. However, we have re-worded this paragraph in the Discussion to better highlight the different approaches that can be taken to estimating the full matrices, using both individual level data (as we have demonstrated in the Methods), and summary level data (using either cross-trait LD score regression or the method proposed by Ray and Boehnke, 2018, which we have included as an additional reference).

“*The proposed approach has some limitations. It uses as input the full covariance matrix of the genetic variant-trait associations. If it assumed that the traits are uncorrelated or that the genetic variant-trait associations are estimated in separate samples, then these matrices can be easily constructed from the standard errors of the genetic association estimates which are typically available from published GWAS results. In practice, it is unlikely that the entire set of traits will be uncorrelated, since they would typically be related at least via common association with the primary trait of interest. We have shown how the full covariance matrices can be estimated using estimates of the trait correlations, either from individual level data or from a reference dataset. Furthermore, the simulation study suggested that, unless the traits are highly correlated with each other, the method is robust to ignoring the genetic variant-trait association correlations. This also suggests that the approach is robust to some participant overlap in the samples. If the traits are highly correlated, there is significant sample overlap, and individual level data are not available, there exist methods to estimate the correlation between genetic associations using summary level data. One approach is to use the intercept term from cross-trait LD score regression [40]. Another is to estimate the correlation between genetic association estimates using only variants which are assumed to not be associated with the traits [41].”*

4. I may have missed but did not find details about sample sizes, the number of significant variants in all, and the number of significant variants after pruning for each of the traits considered from Pulit et al, UK Biobank, etc. I think readers will find these details useful in interpreting results from NAvMix. In particular, I think NAvMix requires large sample sizes and highly polygenic traits.

We have included an extra table in the Supporting Information (Table E) which gives the sample sizes for each trait used in the clustering as well as the number of the BMI associated variants which are significant with the trait at the genome-wide significance level.

5. With respect to Fig 5, the authors mention “The components of BMI predicted by Clusters 1, 2, and 3 show a similar effect size and direction to the overall result.” Does this mean the clusters 1, 2 and 3 are not quite different and could have been just one cluster?

Although these three clusters gave similar results in terms of the effects of their genetically predicted level of BMI on CHD, it may be that they differ in terms of downstream effects with other disease outcomes. Our approach suggests that these clusters of variants are distinct in terms of their biological mechanisms with respect to the chosen traits, although these different mechanisms will not necessarily relate to differences in CHD risk. That said, there is some attenuation to the null of the effect from Cluster 3 variants in comparison to Clusters 1 and 2. This agrees with previous literature that suggests that SBP may mediate some of the effect of BMI on CHD, since Cluster 3 variants do not associate, on average, with SBP.
